# Simulation and Measurement of Strain Waveform under Vibration Using Fiber Bragg Gratings

**DOI:** 10.3390/s24196194

**Published:** 2024-09-25

**Authors:** Nurzhigit Smailov, Sauletbek Koshkinbayev, Bazarbay Aidana, Ainur Kuttybayeva, Yerlan Tashtay, Amir Aziskhan, Dmitry Arseniev, Dmitry Kiesewetter, Sergey Krivosheev, Sergey Magazinov, Victor Malyugin, Changsen Sun

**Affiliations:** 1 Department of Electronics, Telecommunications and Space Technologies, Satbayev University, Almaty 050013, Kazakhstan; n.smailov@satbayev.university (N.S.); a.bazarbay@satbayev.university (B.A.); ainur.kuttybayeva2023@gmail.com (A.K.); y.tashtay@satbayev.university (Y.T.); amirazishan@gmail.com (A.A.); 2 Department of IT and Telecommunications, Miras University, Shymkent 160012, Kazakhstan; sauke49@mail.ru; 3 Peter the Great St. Petersburg Polytechnic University, St. Petersburg 195251, Russia; vicerector.int@spbstu.ru (D.A.); ksi.mgd@gmail.com (S.K.); magazinov_sg@mail.ru (S.M.); vim@spbstu.ru (V.M.); 4 College of Optoelectronic Engineering and Instrumentation Science, Dalian University of Technology, Dalian 116024, China; suncs@dlut.edu.cn

**Keywords:** fiber Bragg grating, sensor, vibration, deformation, strain, digital signal processing

## Abstract

The work is devoted to the consideration of methods for determining the strain of objects using fiber Bragg gratings under a high-frequency vibration or pulsed mechanical action, which is difficult to perform using widespread methods and devices. The methods are based on numerical processing of the time dependence of the radiation power reflected from the fiber Bragg grating at various wavelengths, which makes it possible to measure strain parameters in a wide range of magnitude and frequencies. The efficiency of the proposed methods is demonstrated by numerical simulation. It is shown that it is possible to restore the strain dependence on time in the range ±1000 μϵ or more from simultaneously measured power dependencies reflected by the fiber Bragg grating using common fiber-optic components. The case of sequential registration of reflected radiation power at different wavelengths to determine the probability density of the distribution of the strain values is also considered. The results of signal processing obtained both by numerical simulation and experimentally for the case of a linear vibration are presented. The technical problems of using the proposed methods are discussed.

## 1. Introduction

Many monographs and textbooks, such as [1,2,3,4], articles, reviews in scientific and specialized journals, in particular [5,6,7,8], which contain extensive information on this area, are devoted to the physics of vibrations and methods of their measurement. There are international and national standards for measuring vibration parameters and industrial measuring instruments. The main vibration parameters are amplitude, speed, acceleration, and frequency. Measuring the deformation of machine elements, mechanisms, and building structures under the influence of vibrations is also an important task, but such a task requires other technical solutions, in particular, the use of strain gauge [9,10] or fiber Bragg gratings (FBG) as a vibration sensor.

Fiber Bragg gratings are currently widely used to work in conditions of strong electromagnetic interference caused by pulsed magnetic fields, powerful ultrahigh frequency radiation, radio transmitting devices, and other sources of interference. The demand for FBG sensors is due to their high sensitivity, small dimensions, long service life, resistance to aggressive environments, explosion safety, insensitivity to electromagnetic interference, and the possibility of multiplexing. Currently, FBG sensors are widely used in engineering to measure temperature, deformation parameters, as well as for other applications.

The principle of operation of FBG sensors is based on measuring changes in the resonant wavelength of the grating or the power of the reflected radiation due to changes in the resonant wavelength of the grating and is described in detail in literary reviews and monographs, in particular, in [11,12,13,14,15,16,17,18,19,20,21,22].

To measure temperature, as well as slow deformations, interrogators [11,12,13] are successfully used in conjunction with FBG, in particular, using wavelength scanning, for example, using linear CCD array. For slow processes, the problem of determining the dependence of deformation on time is not related to the measurement speed of the resonant frequencies of the FBG, but to the accuracy of measurements and the interpretation of the resulting dependencies. In a uni-axial approximation, i.e., assuming that there is only stretching or compression of the FBG along one axis at a constant temperature, such a task is not seriously difficult within the technical capabilities of the equipment used. In more detail analysis of peak tracking techniques for FBG sensors is given in the review [12]. However, the practical use of strain sensors based on FBG for various devices may have its own specifics. The theoretical foundations and experience of creating such systems are presented in reviews and scientific articles, for example [11,12,13,14,15,16,17,19,20,21,22].

It is necessary to use other methods, such as those described in [23,24,25,26,27,28,29,30,31,32,33] to register signals with short pulse exposure. However, such methods are difficult to adapt for vibration measurement. So, in [24,25], all measurements were carried our at the same wavelength, which limited the measurement range of the strain value ϵ. The principle of operation of some devices is based on a change in the reflective properties of the FBG when it is destroyed by a propagating detonation wave, which cannot be used to measure the parameters of continuous vibration. The basic principles and methods for measuring vibration parameters using fiber Bragg gratings are summarized in the reviews [22,34,35,36,37,38,39,40], and are also presented in scientific articles, for example, [41,42,43].

Measuring the parameters of high-frequency vibrations using FBG is a certain difficulty, since it is necessary to provide the technical possibility of registering such signals, possibly over a wide range of changes in the magnitude of strain ϵ, as well as determining the ϵ parameters using obtained data. If it is possible to measure the power *p* reflected by FBG radiation simultaneously at several wavelengths λ, the task of determining vibration parameters is to restore the dependence of the strain (relative deformation) value ϵ on time *t* according to several dependencies $p_{m}$, where *m* is the number of the photodetector channel with a central filtration wavelength $\lambda_{m}$. Technically, to implement the method, it is necessary to use either a source with a wide spectrum of radiation or several narrow-band sources simultaneously, with a system of several photodetectors registering radiation at specified wavelengths and operating simultaneously, such as, for example, a WDM (wavelength-division multiplexing) systems. It is necessary to know the type of spectral dependence of the FBG reflection on which the calculation algorithm of ϵ depends to determine the value of ϵ. Dependencies $p_{m}\left(t\right)$ can also be called signals that require digital processing to obtain vibration parameters. The frequency range of measurement p(t) can extend from zero to hundreds of megahertz and above, and the maximum measured magnitude ϵ is determined by the number of channels *m*. The frequency range of measurement of ϵ using the technique discussed below is limited by the condition of uniformity of stretching or compression of the FBG along the entire length assumed in the calculations. If the mechanical effect is such that the specified condition is violated, the model used requires taking into account the heterogeneity of the impact on the FBG.

For stationary vibrations, i.e., vibrations whose parameters do not change over time, various methods and devices can be used. In this paper, we propose the use of a technique based on a sequential change in the wavelength of an optical radiation source, the preservation of FBG signals at different wavelengths recorded at different times, and the determination of vibration parameters based on such a set of signals.

The physical and mathematical basis of the proposed methods, consisting of numerical simulation and theoretical harmonic analysis of signals, is presented below. The description of the experimental implementation of the method and the results confirming the main patterns of the theoretical consideration are also presented.

## 2. Numerical Simulation of Signals for a Fiber Bragg Grating with Trapezoidal Reflection Spectrum

The numerical simulation task consists of modeling the initial dependence of deformation on time with specified parameters, calculating the signal reflected from the FBG at various wavelengths, adding a given noise level to the calculated signals, and restoring the dependence of deformation on time based on the data obtained.

The simulation of ϵ(t) was performed using recurrent formulas [44] for a stationary sequence of numbers $Y_{n}$, where *n* is the number of the sequence element, with a normal distribution, unit variance, and exponential auto-correlation function *C* of the form:

(1) C(n)=Dexp(−αn)cos(βn)

where α is the attenuation decrement, β is the characteristic cyclic frequency, and *D* is the proportionality coefficient depending on the amplitude of vibrations. Accordingly, the sequence of relative strain values is calculated using the formula:

(2) $$\epsilon_{n}=A_{v}Y_{n}$$

where $A_{v}$ is the coefficient determining the vibration amplitude. It is enough to specify only one coefficient $A_{v}$ and set D=1 to model the $Y_{n}$. Since this paper considered a time-dependent process, the elements of the sequence $\epsilon_{n}$ could be represented as the amount of strain at discrete time points $t_{n}$: $\epsilon (t_{n})$.

**Figure 1:**
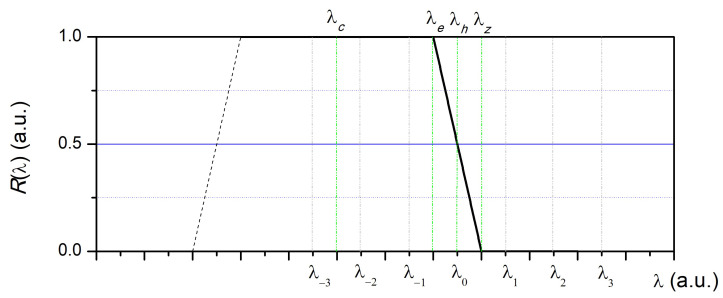
The reflection spectrum of the fiber Bragg grating adopted in the model and the designations of the wavelengths.

Let us consider the simplest model describing the normalized dependence ($R_{f}$) of the power of reflected radiation by a fiber Bragg grating on the wavelength of radiation λ in the operating wavelength range in the following form (Figure 1) by analogy with the dependence presented in [45]:

(3) $$R_{f}\left(\lambda \right)=\left\{\begin{array}{cc} 1 & \;\mathrm{if}\;\lambda <\lambda_{e}\\ 1-\gamma (\lambda -\lambda_{e}) & \;\mathrm{if}\;\lambda_{e}\leq \lambda <\lambda_{e}+\Delta_{sFBG}\\ 0 & \;\mathrm{if}\;\lambda \geq \lambda_{e}+\Delta_{sFBG} \end{array}\right.$$

where (Figure 1) $\lambda_{e}$ is the wavelength corresponding to the beginning of the inclined $R_{f}\left(\lambda \right)$ dependence, $\Delta_{sFBG}$ is the spectral width of the inclined section of the $R_{f}\left(\lambda \right)$, γ is the proportionality coefficient ($\gamma =1/\Delta_{sFBG}$), $\lambda_{c}$ is the resonant wavelength of the FBG, and $\lambda_{z}$ is the wavelength corresponding to the end of the inclined section ($\Delta_{sFBG}=\lambda_{z}-\lambda_{e}$). If the transmission spectrum width of the photodetector filter (or the spectrum width of the radiation source, if, for example, several semiconductor lasers are used) is significantly less than the value $\Delta_{sFBG}$, then the $R_{f}$ dependence is actually the dependence of the power *p* of the radiation reflected by the FBG, normalized to the maximum value, on the wavelength λ.

It is known that the displacement of the resonant wavelength FBG under tension is proportional to the magnitude of the relative FBG deformation (i.e., FBG strain) ϵ. We assume that at the deformations considered below—uni-axial tension or compression—the shape of the $R_{f}$ spectral dependence does not change, i.e., it takes place as follows:

(4) $$\lambda_{c}=\lambda_{c0}+k_{\epsilon }\epsilon ,\lambda_{e}=\lambda_{e}+k_{\epsilon }\epsilon ,\gamma =const\left(\epsilon \right)$$

where $k_{\epsilon }$ is the proportionality coefficient ($k_{\epsilon }\approx 10^{-3}$ μϵ/nm at λ=1310 nm, and $k_{\epsilon }\approx 1.2\times 10^{-3}$ μϵ/nm at λ=1550 nm). That is, when the FBG is stretched, the $R_{f}$ dependence shifts towards longer wavelengths (in Figure 1—to the right along the 0× axis).

Let us choose the wavelengths of radiation $\lambda_{m}$ at which the dependencies of the reflected radiation power on time will be most characteristic for considering the basic patterns. At ϵ=0, let $\lambda_{0}$ be such that $R_{f}\left(\lambda_{0}\right)=1/2$, $\epsilon (\lambda_{0})=0$, and $\lambda_{1}=\lambda_{0}+\Delta \lambda_{sFBG}$, respectively, $\lambda_{2}=\lambda_{0}+2\Delta \lambda_{sFBG}$, …, $\lambda_{m}=\lambda_{0}+m\Delta \lambda_{sFBG}$, $\lambda_{-1}=\lambda_{0}-\Delta \lambda_{sFBG}$, $\lambda_{-2}=\lambda_{0}-2\Delta \lambda_{sFBG}$, …, $\lambda_{-m}=\lambda_{0}-m\Delta \lambda_{sFBG}$, where m={…,−2,−1,0,1,2,…} is an integer. Then, Formula (3) can also be represented as:

(5) $$R_{f}\left(\epsilon \right)=\left\{\begin{array}{cc} 1 & \;\mathrm{if}\;\lambda_{m}\leq \lambda_{e}\\ 1/2+\gamma (k_{\epsilon }\epsilon +\lambda_{h}-\lambda_{m}) & \;\mathrm{if}\;\lambda_{e}\leq \lambda_{m}<\lambda_{z}\\ 0 & \;\mathrm{if}\;\lambda_{m}\geq \lambda_{z} \end{array}\right.$$

where $\lambda_{h}$ is the central wavelength of the falling section, at which the equality $R_{f}\left(\lambda_{h}\right)=1/2$ is satisfied, i.e., $\lambda_{0}=\lambda_{h}$. The dependence ϵ(t) is given in the form of a series of numbers $\epsilon (t_{n})$ generated according to the above algorithm in accordance with Formulas (1) and (2).

For small deformations, i.e., if the inequality $k_{\epsilon }\epsilon <\Delta \lambda_{sFBG}/2$ and $\lambda_{m}=\lambda_{h}$ always holds:

(6) $$\epsilon \left(t\right)=\left(R_{f}\left(t\right)-1/2\right)/\left(\gamma k_{\epsilon }\right)$$

Thus, with a small deformation, no special algorithms need to be used to determine the dependence of ϵ(t); it is enough to perform a measurement at one wavelength ($\lambda_{h}$), since the magnitude of ϵ is proportional to the amplitude of the reflected FBG signal. The linear elongation of the FBG leads to a linear increase in the reflected FBG power *p* until *p* reaches its maximum value (i.e., $R_{f}=1$). When FBG is compressed, the power *p* is reduced to zero (i.e., $R_{f}=0$).

If the vibration amplitude is such that $k_{\epsilon }\epsilon >\Delta \lambda_{sFBG}/2$, measurements must be performed simultaneously at several wavelengths. Taking into account that the measurement range at one wavelength is $\Delta \lambda_{sFBG}$, it is advisable to select the measurement wavelengths with an offset by the specified amount $\Delta \lambda_{sFBG}$, as indicated above. Then, the magnitude of the strain ϵ(t) can be determined from the sections of dependencies p(t) specified at points $t_{n}$, at which the dependence has a non-zero and non-equal-to-one value, as well as assuming the same spectral sensitivity:

(7) $$\epsilon \left(t_{n}\right)=\left\{\begin{array}{cc} 0 & \;\mathrm{if}\;R_{f}\left(t_{n}\right)=0\\ \sum_{j=-m}^{m}\left(\lambda_{j}-\lambda_{h}+\left(R_{f}\left(t_{n}\right)-1/2\right)/\gamma \right)/k_{\epsilon }) & \;\mathrm{if}\;0<R_{f}\left(t_{n}\right)<1\\ 0 & \;\mathrm{if}\;R_{f}\left(t_{n}\right)=1 \end{array}\right.$$

The described technique and Formula (7) assume the idealized dependence $R_{f}\left(\lambda \right)$. When processing experimental data, it is necessary to take into account both the noise level and the deviation of the $R_{f}\left(\lambda \right)$ dependence from 1 in the range $\lambda <\lambda_{h}-\Delta_{sFBG}/2$, for example, excluding points near 0 and 1 from consideration and, if necessary, using wavelength offset $\lambda_{m}$ less than $\Delta \lambda_{sFBG}$.

**Figure 2:**
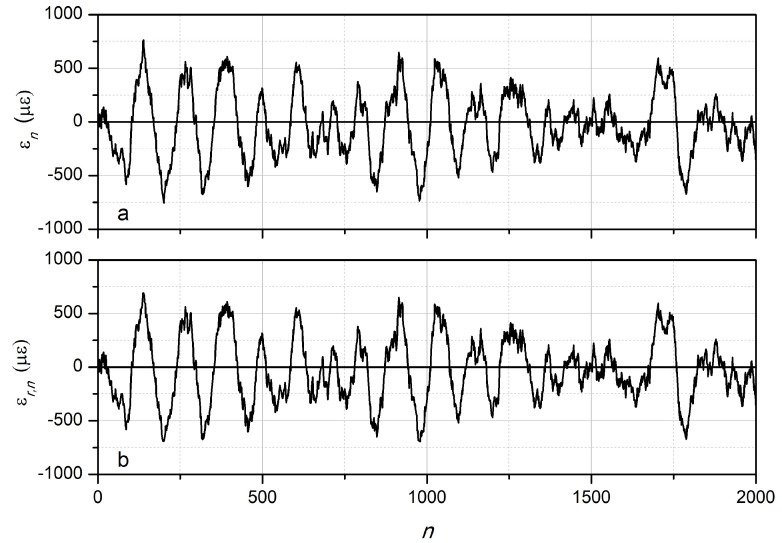
Numerical modeling of the dependencies of the strain of the FBG on time in the form of the sequence of samples *n*: (**a**)—given using recurrent formulas, (**b**)—reconstructed from seven dependencies of the radiation power reflected by the FBG.

**Figure 3:**
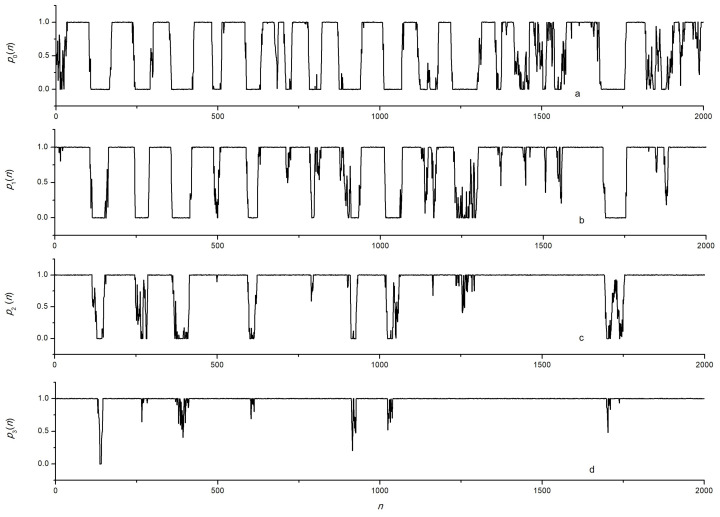
Waveform of signals obtained by numerical simulation for channels *m*: (**a**)—0, (**b**)—1, (**c**)—2, (**d**)—3.

**Figure 4:**
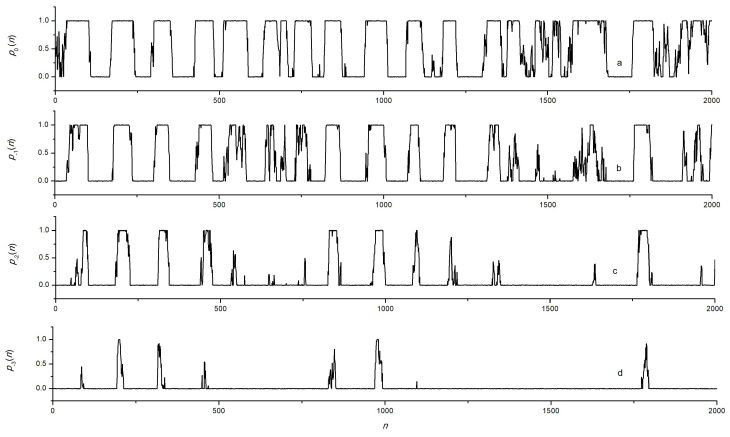
Waveform of signals obtained by numerical simulation for channels *m*: (**a**)—0, (**b**)—−1, (**c**)—−2, (**d**)—−3.

As an example, the modeling of signals recorded at various wavelengths with the following parameters was performed: $Y_{n}$ was a sequence of numbers with normal distribution, whose sequence parameters were given by Formulas (1) and (2): α=1/150, β=1/20, $A_{v}=300$, at $\lambda_{0}=\lambda_{c0}=1310$ nm, $k_{\epsilon }\approx 1.014\times 10^{-3}$ μϵ per nm, $\Delta \lambda_{sFBG}$ = 0.2 nm, γ=5. A random noise of 1% was added to the signal calculated by Formula (2). The specific sampling value $\Delta \tau =t_{n+1}-t_{n}$ does not matter in this case, and all time dependencies are given later in the article as dependencies on the reference number. An example of the simulated initial dependence ϵ(t) obtained by interpolating the values of $\epsilon_{n}$ is shown in Figure 2a. The dependencies of the normalized power p(t) for seven wavelengths are shown in Figure 3 and Figure 4.

Let us consider the entity and basic patterns of the dependencies shown in Figure 3 and Figure 4. On the first flat section $p_{0}\left(n\right)$ for $\lambda_{0}$, extending approximately from number 35 to number 100, there is a stretching of the FBG, leading to $p_{0}\left(n\right)=1$. Moreover, at wavelengths $\lambda_{-1}$, $\lambda_{-2}$, and $\lambda_{-3}$, $p_{m}\left(n\right)=1$ occurs due to $p_{0}\left(n\right)=1$ (Figure 4). At large wavelengths $\lambda_{1}$, $\lambda_{2}$, and $\lambda_{3}$, the value of $p_{m}\left(n\right)$ in this range *n* sometimes reaches 0, and at $\lambda_{3}$ it becomes different from 0 only at the moments of the largest stretching of the FBG (Figure 3). The range of *n* is from 110 to 160 (Figure 3 and Figure 4, curves “a”) corresponds to the compression of the FBG at which $p_{0}\left(n\right)=0$. At wavelengths $\lambda_{-1}$, $\lambda_{-2}$, and $\lambda_{-3}$, the value of $p_{m}\left(n\right)$ in this range sometimes reaches 1, and at $\lambda_{-3}$, it becomes different from 1 only at the moments of the greatest compression of the FBG (Figure 4).

The dependence ϵ(t), reconstructed from the seven dependencies $p_{m}\left(t\right)$ at different wavelengths, is shown in Figure 2b. As follows from Figure 2, the restored dependence $\epsilon_{r,n}$ almost coincides with the original dependence $\epsilon_{n}$ even with the noise level of 1% introduced.

## 3. Numerical Simulation of Signals for a Fiber Bragg Grating with Gaussian Reflection Spectrum

The fundamental difference between the signals of the FBG with a Gaussian reflection spectrum from those previously considered is due to the fact that, unlike the trapezoidal spectrum, any normalized value not equal to one of the reflected power always corresponds to two different values of strain of the FBG, as well as the fact that the signal can be zero only, not equal to zero, and one, as for trapezoidal spectrum, at a deformation of the FBG outside the range of Gaussian reflection.

The waveform of the pulse deformation in the FBG in the case of a Gaussian spectrum is considered in [24,25]. It follows that the type of pulse signal, and even more so its parameters, depend on the difference in the wavelengths of the radiation source and the resonant wavelength of the reflection of the FBG.

The main properties of the vibration signal reflected from the FBG can be explained based on the analysis of signals with harmonic deformations of the FBG at various operating points (i.e., at different wavelength differences). Let

(8) $$\epsilon \left(t\right)=A_{\epsilon }\sin\left(\omega t\right)$$

where $A_{\epsilon }$ is the magnitude of the strain of the FBG at the vibration, ω is the cyclic deformation frequency, and *t* is the time. Then,

(9) $$p\left(t\right)=A_{p}\exp\left(-{\left(\frac{k_{\epsilon }\epsilon \left(t\right)-\Delta \lambda_{s}}{\sigma_{s}}\right)}^{2}\right)$$

where $A_{p}$ is the proportionality coefficient, $\sigma_{s}$ is the equivalent half–width of the reflection spectrum of the FBG [24,25], $\Delta \lambda_{s}=\lambda_{m}-\lambda_{FBG0}$ is the difference between the wavelengths of the radiation source and the resonant wavelength of the FBG at zero deformation (i.e., in the absence of stretching or compression). Let us further consider the normalized functions p(t), i.e., assuming $A_{p}=1$.

**Figure 5:**
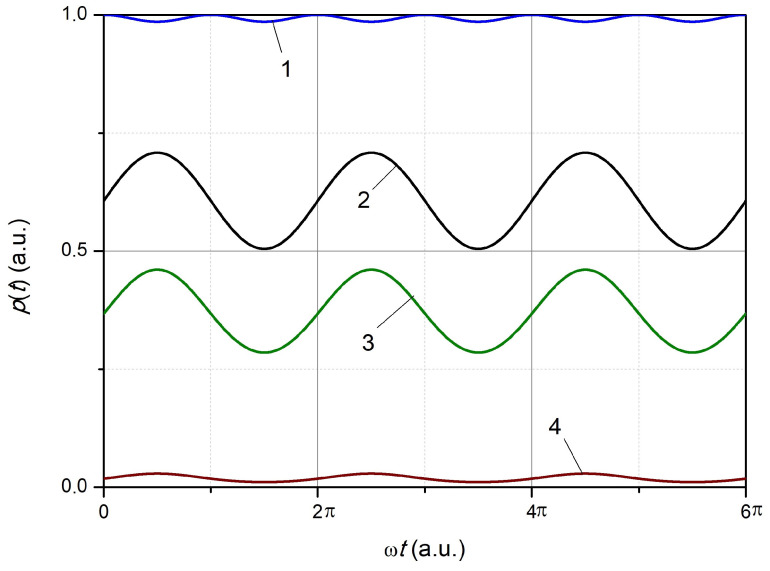
The dependence of the power of the reflected FBG radiation on the oscillation phase during a periodic deformation with $A_{\epsilon }=10$ and various normalized wavelength difference $\Delta \lambda_{s}$: 1—0, 2—$\Delta \lambda_{s}=-\sigma_{s}/2$, 3—$\Delta \lambda_{s}=-\sigma_{s}$, 4—$\Delta \lambda_{s}=-2\sigma_{s}$.

First, let us consider the case of a weak vibration ($A_{\epsilon }\ll \sigma_{s}/k_{\epsilon }$). An example of the calculated dependencies of p(t) at four characteristic points, at the top of the spectrum (at $\lambda_{m}=\lambda_{FBG0}$, i.e., at $\Delta \lambda_{s}=0$), on the slope of the spectrum ($\Delta \lambda_{s}\neq 0$, $|\Delta \lambda_{s}|\leq \sigma_{s}$), and in the region of small values of the reflected power ($|\Delta \lambda_{s}|>\sigma_{s}$), is shown in Figure 5. It follows from the obtained dependencies that at the top of the spectrum the reflected signal (Figure 5, dependence 1) has a doubled frequency compared to the deformation frequency and a relatively small amplitude. The signals have a shape close to sinusoidal but different amplitudes (Figure 5, dependencies 2 and 3) on the slope of the spectral dependence at the point of maximum sensitivity ($\Delta \lambda_{s}=-\sigma_{s}/2^{1/2}$) and slightly lower. The signal has a shape similar to the above dependencies but a significantly lower amplitude at $\Delta \lambda_{s}<-2\sigma_{s}$. The phase of the reflected signal on a slope with $\Delta \lambda_{s}<0$ coincides with the phase of strain oscillations, and on a slope with $\Delta \lambda_{s}>0$, it has the opposite phase.

A characteristic of this phenomenon is the dependence of the harmonic coefficient of the signal on the position of the working point (i.e., the difference $\Delta \lambda_{s}$). The harmonic coefficients were calculated using formulas similar to the calculation of nonlinear distortion coefficients:

(10) $$k_{n}=\frac{{\left(a_{n}^{2}+b_{n}^{2}\right)}^{1/2}}{{\left(\sum_{n=1}^{\infty }\left(a_{n}^{2}+b_{n}^{2}\right)\right)}^{1/2}}$$

where

(11) $$a_{n}=\frac{1}{\pi }\int_{0}^{2\pi }p\left(t\right)\cos\left(n\omega t\right)dt$$

(12) $$b_{n}=\frac{1}{\pi }\int_{0}^{2\pi }p\left(t\right)\sin\left(n\omega t\right)dt$$

*n* is the harmonic number in Formulas (10)–(12).

For the considered examples, the values of the fourth and higher harmonics were neglected. Trends $k_{1}\to 0$ and $k_{2}\to 1$ took place when $\Delta \lambda_{s}=0$ and $A_{\epsilon }\to 0$.

**Figure 6:**
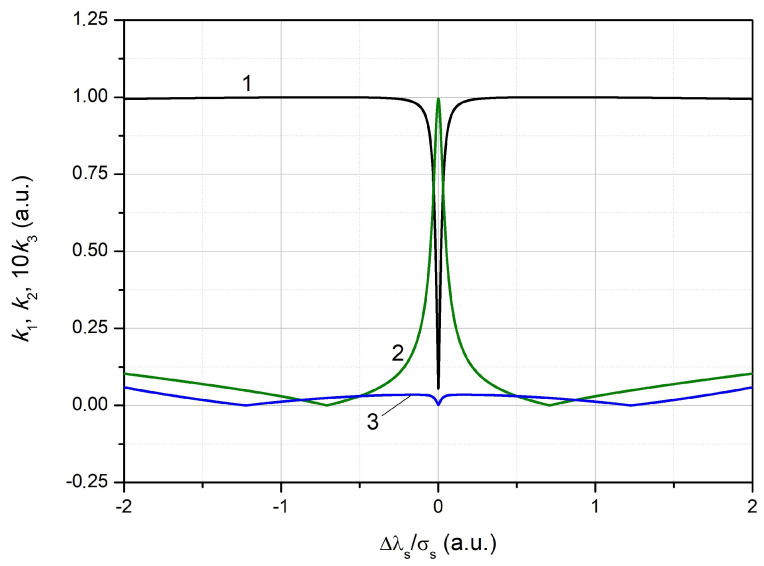
The dependence of the harmonic coefficient of the signal caused by the reflected radiation from the FBG on the normalized wavelength difference: 1—the first harmonic, 2—the second, 3—the third (on the graph, the harmonic value is increased 10 times).

The example of the calculated dependencies is shown in Figure 6.

**Figure 7:**
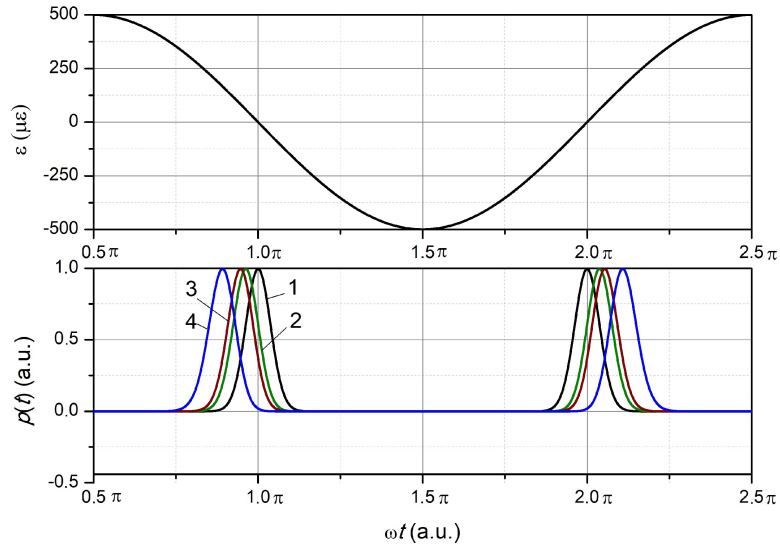
Dependencies of magnitude of the strain and power of the reflected radiation on the phase of deformation oscillations at $A_{\epsilon }=500$ and various normalized wavelength differences $\Delta \lambda_{s}$: 1—$\Delta \lambda_{s}=0$, 2—$\Delta \lambda_{s}=-\sigma_{s}/2$, 3—$\Delta \lambda_{s}=-\sigma_{s}$, 4—$\Delta \lambda_{s}=-2\sigma_{s}$.

For a strong periodic deformation, i.e., $A_{\epsilon }\gg \sigma_{s}/k_{\sigma }$, the shape of the reflected FBG signal is close to Gaussian with an average period equal to the half-life of the FBG deformation (Figure 7).

As follows from the data obtained (Figure 7), when compressing the FBG (the interval from π/2 to 3π/2 in Figure 7) first, the pulse appears at $\Delta \lambda =-2\sigma_{s}$, then at $\Delta \lambda =-\sigma_{s}$ and $\Delta \lambda =-\sigma_{s}/2^{1/2}$. Dependence 1 (Figure 7) corresponds to the absence of deformation of the FBG, which occurs at the oscillation phase ωt equal to π. When the FBG is compressed, the specified pulse is the last of the specified ones. The pulse sequence is reversed when stretching the FBG (the interval from 3π/2 to 5π/2 in Figure 7). The greater the value of $A_{\epsilon }$, the shorter the duration of Gaussian-like pulses. The time interval between pulses is always equal to the value π/ω at Δλ=0, greater than π/ω in the range of transition from compression to tension at Δλ<0, and less than π/ω in the range of transition from tension to compression (not shown in Figure 7).

Based on the studied patterns, it can be concluded that the method of signal reconstruction based on time dependencies measured simultaneously at several wavelengths requires changes compared to the previously considered method for the trapezoidal spectral dependence of the FBG. First, it is advisable to exclude measurements for m=0 (i.e., for $\Delta \lambda_{0}$) in order to avoid the difficulty of unambiguously determining deformation by choosing an even non-zero number of wavelengths, for example, as $\Delta \lambda_{\pm 1}=\pm \sigma_{s}/2$, $\Delta \lambda_{\pm 2}=\pm 3\sigma_{s}/2$, $\Delta \lambda_{\pm 3}=\pm 5\sigma_{s}/2$, …, $\Delta \lambda_{\pm m}=\pm \left(2m-1\right)\sigma_{s}/2$. The number of wavelengths at which the measurement is performed (and, accordingly, the number of photodetectors) should be such that the radiation reflected from the FBG during the measurement process is always recorded by some photodetector.

**Figure 8:**
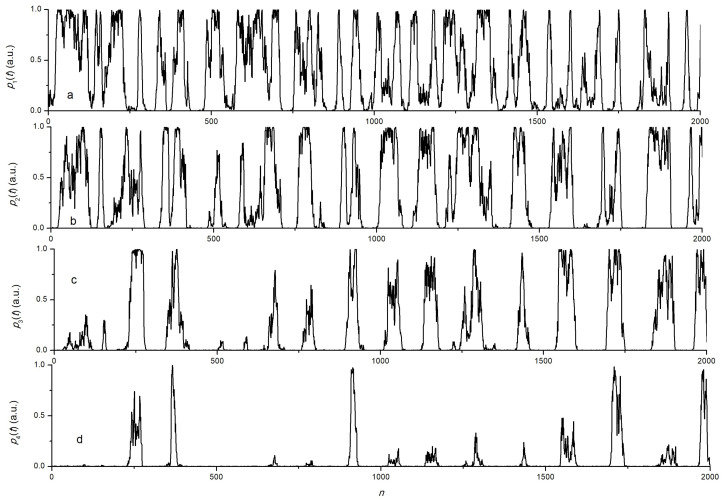
An example of waveform obtained by numerical simulation for the wavelength differences $\Delta \lambda_{s}$: (**a**)—$\Delta \lambda_{s}=\sigma_{s}/2$, (**b**)—$\Delta \lambda_{s}=3\sigma_{s}/2$, (**c**)—$\Delta \lambda_{s}=5\sigma_{s}$, (**d**)—$\Delta \lambda_{s}=7\sigma_{s}$.

An example of the simulated dependencies of the power, simultaneously measured at different wavelengths, is shown in Figure 8. The example illustrates the patterns of signals described above. The signal was restored using dependencies $p(\lambda_{-4})$, $p(\lambda_{-3})$, …, $p(\lambda_{3})$, $p(\lambda_{4})$, starting from the dependence $p(\lambda_{4})$ according to the following algorithm. We assumed that the change in the wavelength of the FBG under the action of vibrations was in the range of the possibility of registering a signal by photodetectors at these wavelengths or, at least, at the initial section of the function $p(\lambda_{4})$. Let us assume that the reliably recorded power value in each channel, taking into account noise, is, for example, in the range $0.05<p_{m}<0.95$. Starting from n=1 for each point in the sequence $p_{m}\left(n\right)$ power values, we checked the fulfillment of the condition:

(13) $$0.05<p_{m}\left(n\right)<0.95$$

for all channels *m*, starting from m=−4 (i.e., $p_{-4})$. If the condition (13) was fulfilled, then the value of the strain was calculated by the formula:

(14) $$\epsilon_{n}=\left(-\sigma_{s}{\left(-\ln\left(p_{m}\left(n\right)\right)\right)}^{1/2}+\Delta \lambda_{s}\right)/k_{e}$$

**Figure 9:**
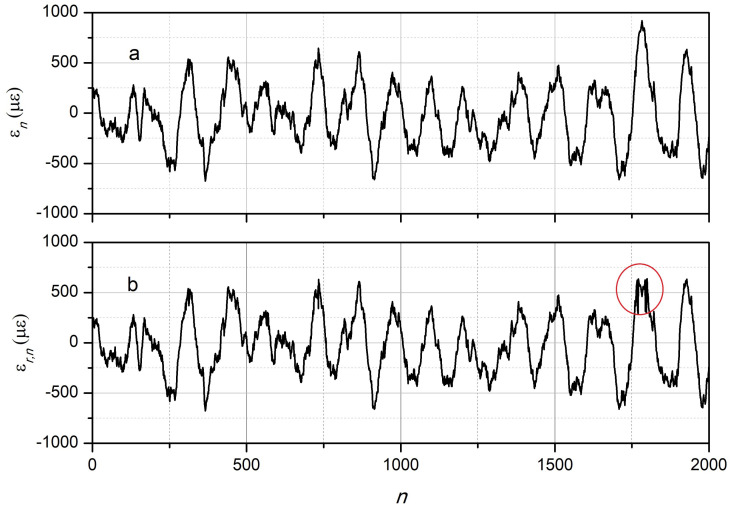
The initial (**a**) and reconstructed (**b**) dependence of the strain on the sample number.

For the remaining wavelengths of $\lambda_{m}$, the value of $\varepsilon_{n}$ was not calculated. Random noise in the amount of 1% of the maximum value was added to the $p_{m}$ signals of each channel. An example of the initial waveform of strain ($\epsilon_{n}$) of the FBG during vibration and the resulting waveform restoration ($\epsilon_{n,r}$) using eight wavelengths is shown in Figure 9. As follows from Figure 9, there was a good coincidence of the dependencies $\epsilon_{n}$ and $\epsilon_{n,r}$. Note also that in the presented example, a fragment of the dependence $\epsilon_{n}$ was specially selected for which, in the range *n* from 1773 to 1793, the value of ϵ exceeded 750 μϵ, which made it impossible to accurately restore the signal in this *n* range. Therefore, if necessary, in similar cases, for example, to identify the maximum values of stretching or compression of the FBG during vibration, it is necessary to add another measurement channel at the wavelength of $\lambda_{-5}$ for which $\Delta \lambda_{-5}=-9\sigma_{s}/2$.

Thus, the sequence of actions for determining the dependence of ϵ(t) is as follows. Starting from the first element (n=1) of the $p_{m}$ samples, $p_{m}\left(n\right)$ values are scanned by the index *m*, starting from the first or last value of *m*. In the above example, processing was performed from m=−4 to m=4. Condition (13) is checked for each index *m*. If the condition is met, the ϵ value is calculated for this sequence of $p_{m}$ (i.e., for indices *m* and *n*) using Formula (14). Further scanning by the index *m* is interrupted, the verification of condition (13) is started anew for the next number of the sequence element (n+1) from the first or last value of *m*. The calculation is completed when the last element of the $p_{m}\left(n\right)$ samples is reached.

Taking into account the ranges for determining the amount of deformation overlap, the accuracy of the signal recovery can be improved by choosing a range in which this value of deformation is calculated with greater accuracy. It can be assumed that the accuracy of the determination of ϵ is proportional to the modulus of the derivative of the dependence p(ϵ), determined by Formula (9), with respect to ϵ. At $A_{\epsilon }\to 0$, the maximum is reached at $\Delta \lambda_{s}=\pm \sigma_{s}/2^{1/2}$ [24,25], and in the case under consideration, it is advisable to choose an array $p_{m}$ in which the modulus of the derivative p(ϵ) at this point (*n*) is greater.

## 4. Determination of Vibration Parameters by Samples with Successive Changes in the Wavelength

In many cases, it is not technically possible to measure the power of the reflected radiation simultaneously at several wavelengths. If vibration is a stationary process, i.e., the vibration parameters are constant at least during the measurement, then, in that case, the density distribution of the strain can be determined from samples of signals measured sequentially at different wavelengths at different times. Technically, such measurement can be performed using a semiconductor laser mounted on a Peltier element, which allows the temperature of the laser heterojunction to be changed, as well as the wavelength of the radiation. The result of such measurement is *m* fragments of power versus time, measured sequentially at *m* wavelengths. Naturally, it is impossible to restore the full dependence of ϵ(t) in this case, but the density distribution of ϵ(t) calculated from *m* fragments will correspond to the distribution density of the full ϵ(t).

In contrast to the used signal reconstruction technique described earlier for the Gaussian spectral dependence of the FBG reflection, in this case, it is impossible to use the exclusion of samples at other wavelengths after determining ϵ at any point in one of the samples. Therefore, it is necessary to exclude in another way the influence of overlapping areas of the $p_{m}\left(\epsilon \right)$ dependencies on the result of calculating the probabilistic characteristics of the process. This is achieved by selecting the signal levels in each channel, within which the value of ϵ is determined; thus, the end of one range in one channel is the beginning of the measurement range in another channel (for example, in channels $p_{m}$ and $p_{m+1}$). Let $u_{m,l}$, $u_{m,p}$ be the lower and upper limits of the range of determination of ϵ. The wavelength shift between the channels is equal to the value $\sigma_{s}$ when:

(15) $$u_{m+1,l}=\exp{\left(-{\left(-\ln\left(u_{m,l}\right)\right)}^{1/2}-1\right)}^{2})$$

If the upper level (the upper limit of the measurement range in channel *m*) is set as 0.95 of the maximum value, then when the wavelengths are shifted by the value of $\sigma_{s}$, the corresponding lower level in the adjacent channel (m+1) will be approximately equal to 0.22218. Accordingly, the calculation range of ϵ used to calculate the probabilistic characteristics will be from 0.22218 to 0.95.

The method was verified by numerical modeling for the Gaussian distribution $\epsilon (t_{n})$ obtained using the previously described technique with the Gaussian FBG reflection spectrum. The simulation result is described below.

## 5. Simulation of Determination of the Probability Density of the Strain Distribution with a Sequential Change in the Wavelength of the Radiation

If there is no technical possibility of simultaneous measurement of the reflected radiation at different wavelengths, then for a stationary process (i.e., with constant statistical parameters), the probability density of the strain distribution can be determined by a sequential change in the wavelength of the source radiation. Technically, this can be accomplished either by changing the wavelength of the laser or using a tunable spectral filter for the reflected FBG radiation at a broadband radiation source. Since in this case, at each wavelength, the dependencies p(t) are obtained sequentially at different points in time, it is impossible to restore the dependence ϵ(t). However, due to the stationarity of the random process ϵ(t), it is sufficient to determine the probability density of the distribution ϵ(t) in the operating range of the measured values ϵ for the dependencies p(t) at each wavelength, so that the measurement ranges ϵ do not overlap.

This technique was tested by numerical simulation for the case of reflected FBG radiation with a Gaussian spectrum and the Gaussian distribution ϵ(n). Using the method described earlier and Formula (14), 10 arrays of 4096 numbers each of $Y_{n}$ values with the λ offset from $-9\sigma_{s}/2$ to $+9\sigma_{s}/2$ were generated. Then, the corresponding dependencies of $p_{m}\left(n\right)$ were calculated at different wavelengths with a shift by the value of $\sigma_{s}$. The coefficient $A_{\epsilon }$ was set to 300, $k_{\epsilon }$ was $1.014\times 10^{-3}$ nm/μϵ, and $\sigma_{s}$ was 0.2 nm. For each array, the values of ϵ corresponding to the elements of *p* were determined. A random noise of 1% of the maximum value of *p* (in this case – 1) was artificially added to all the obtained values of the $p_{m}\left(n\right)$ elements. Further, similar to the above-described technique for the case of the Gaussian reflection spectrum of the FBG, the values of ϵ in each $p_{m}$ array were determined. The obtained values of ϵ were combined into one array and, using a standard statistical data processing program, a histogram of the distribution of ϵ was obtained. The difference in the calculated histogram of the distribution ϵ(t) obtained from 10 fragments and the histogram of the initial dependence ϵ(t) was no more than 5%. The average shape of the histogram approached the Gaussian one, and the existing difference was due to a statistical error when the numerical experiment was repeated many times. The result can be considered expected, since it was initially assumed that the random process was stationary. Therefore, an example of the resulting histogram is not provided.

## 6. Experiment

**Figure 10:**
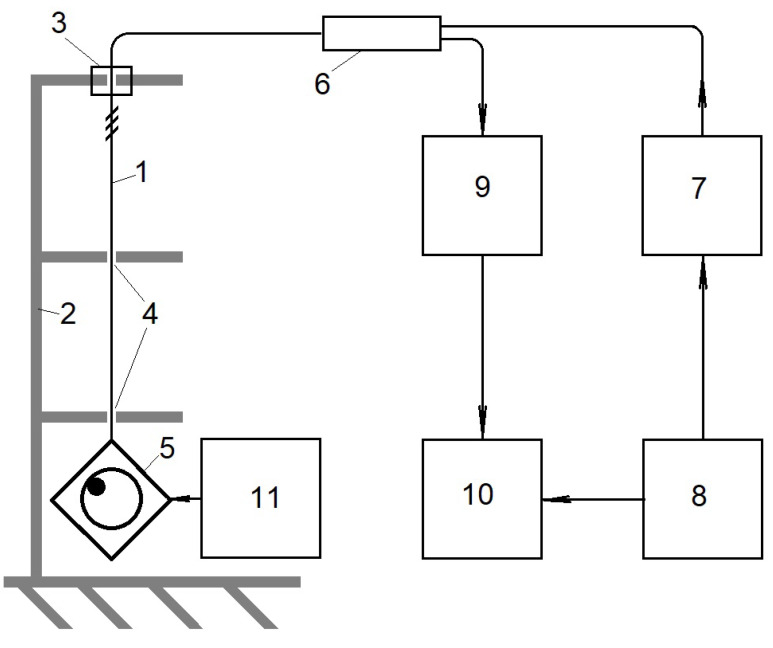
The scheme of the experimental installation: 1—optical fiber with FBG, 2—mechanical construction, 3—device fixing the optical fiber, 4—holes in the brackets, 5—electric motor with an eccentricity, 6—fiber optic splitter, 7—semiconductor laser with Peltier element, 8—laser temperature control unit, 9—photodetector module, 10—personal computer with ADC converter, 11—motor power supply.

The experimental study was performed on a single-mode optical fiber with a fiber Bragg grating with a central wavelength at room temperature of 1310 nm, a reflection spectrum width of 0.0777 nm ($\sigma_{FBG}=0.0462$ nm), an FBG length of 15 mm, with a maximum reflection coefficient of 90%. The scheme of the experimental installation is shown in Figure 10. The optical fiber 1 with the fiber Bragg grating was fixed in the upright position on the special mechanical construction 2 by a holder 3. The electric motor 5 with an eccentricity was attached to the optical fiber from below. The optical fiber was passed through two holes 4 in the mounting brackets between FBG and the electric motor. The diameter of the holes 4 exceeded the outer diameter of the fiber sheath by about 500 μm. The distance between the brackets was approximately 0.5 m, and that between the FBG and the electric motor was −1 m. The installation design made it possible to practically eliminate the bends of the FBG during vibration, which allowed us to assume the vibration of the FBG to be uni-axial.

The DFB-type semiconductor laser 7 with a wavelength of 1310 nm, a half-width of the radiation spectrum $\sigma_{LD}$ equal to 0.015 nm, and a temperature coefficient of wavelength variation of 0.071 nm/°C was used as a radiation source. The laser was mounted on the Peltier element, which made it possible to change the temperature of the laser heterojunction within ±15 °C by changing the amplitude and direction of the current flowing through the element, using the power supply 11. The possible wavelength adjustment exceeded 2 nm. The accuracy of the laser temperature measurement was approximately 0.1 °C. The equivalent value of the half-width of the $\sigma_{s}$ spectrum used in the calculations was set to 0.05 nm. The change in the laser temperature, leading to the specified value of the wavelength offset by the value $\sigma_{s}$, was 0.7 °C.

The dependencies of p(t) were determined using standard passive fiber-optic components—photodetector 9 and splitter 6 with a 50%/50% division coefficient according to the standard scheme in [25] for measurements of the power of radiation reflected from the FBG. The signal from the photodetector module was transmitted to the analog-to-digital converter and stored in the memory of a personal computer 10. The sampling frequency was 100 kHz, the arrays contained approximately $10^{6}$ samples each; however, only 4096 array elements with the specified sampling interval were used to calculate the distribution parameters. The optical fiber with the fiber Bragg grating was fixed in the upright position. An electric motor with an eccentricity was mounted at the bottom of the fiber with the FBG, creating uni-axial vibrations in the FBG, the shape of which could be assumed to be sinusoidal. The resonant wavelength of the fiber Bragg grating, elongated by the weight of the vibrating device in the absence of vibration, was taken as the initial wavelength $\lambda_{FBG0}$.

**Figure 11:**
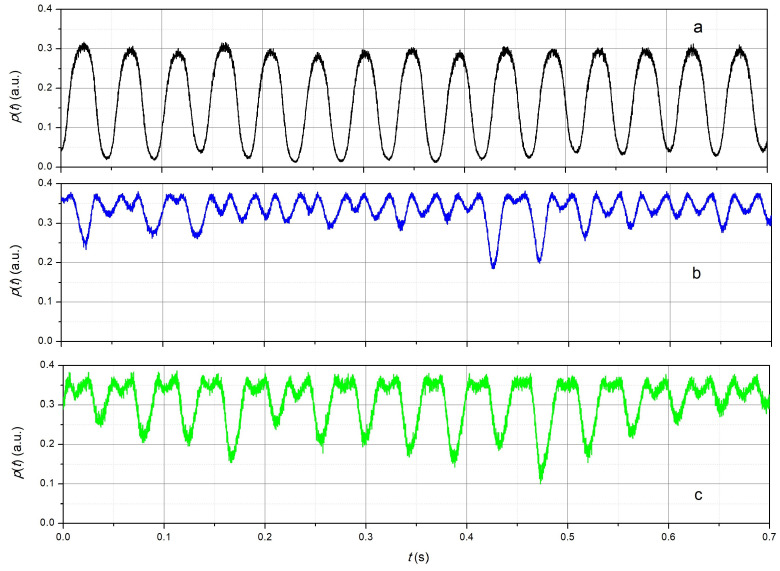
Dependencies of the power of the radiation reflected by the fiber Bragg grating on time at different wavelengths: (**a**)—$\lambda_{LD}\approx \lambda_{FBG0}-\sigma_{s}/2^{1/2}$, (**b**)—$\lambda_{LD}\approx \lambda_{FBG0}$, (**c**)—$\lambda_{LD}∼\lambda_{FBG0}$.

**Figure 12:**
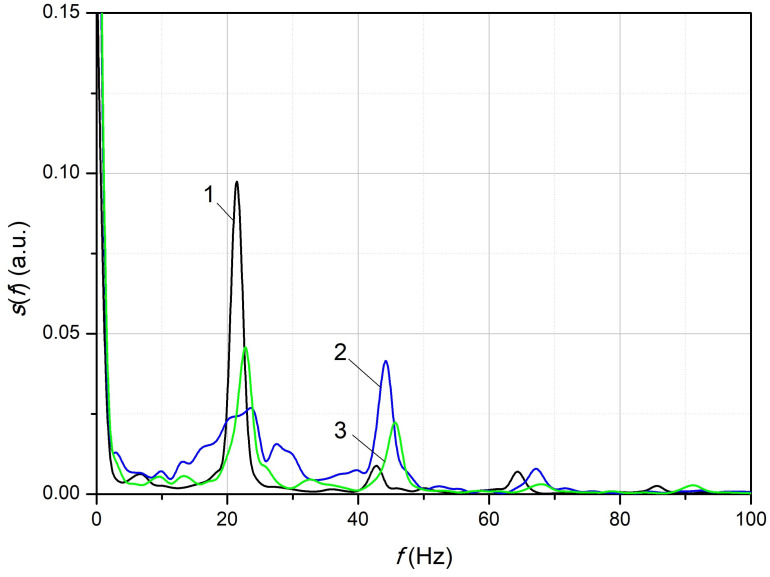
The spectral density of the signals, the waveform of which are shown in Figure 11: 1—(a), 2—(b), 3—(c).

The p(t) dependencies obtained at different laser temperatures with a step of 0.1 °C were preserved. An example of the obtained dependencies of p(t) for a relatively small deformation is shown in Figure 11. Figure 11a corresponds to the case of the location of the working point near the sensitivity maximum, b—at the maximum, i.e., approximately at $\lambda_{LD}=\lambda_{FBG0}$, c—near the maximum of the reflection spectrum of the FBG. The characteristic properties of the obtained dependencies corresponded to the results obtained by numerical simulation for harmonic oscillations (Figure 5). The dependence p(t) in Figure 11a had a shape close to sinusoidal with a period of approximately 46 ms, which corresponds to a frequency of 22 Hz. The dependence p(t) shown in Figure 11c was due to the fact that the resonant wavelength of the FBG, which varied with the vibration, reached the wavelength of radiation of the source and exceeded it for some time. This led to a noticeable appearance of the second harmonic of the vibration signal. Figure 11b corresponds to the case $\lambda_{LD}=\lambda_{FBG0}$ in which the signal p(t) had a doubled vibration frequency of approximately 44 Hz. The estimated value of the vibration magnitude, as shown in Figure 11a, was approximately 30μϵ. The spectral density of the p(t) signals shown in Figure 11 were calculated (Figure 12). It followed from the obtained dependencies (Figure 12) that in the first case (dependence a, Figure 11), the first harmonic of the signal was dominant. The maximum spectral density of the second ($s_{max,2}$) and third ($s_{max,3}$) harmonics was approximately 11 and 14 times less than the first ($s_{max,1}$), respectively. In the second case (dependence b, Figure 11), the second harmonic dominated, in which $s_{max,1}$ and $s_{max,3}$ were 1.5 and 3 times smaller than $s_{max,2}$. In the third case (dependence c, Figure 11), $s_{max,2}$ and $s_{max,3}$ were 2 and 16 times smaller than $s_{max,1}$. That is, the experimental data obtained confirmed the identified characteristic features of the signals generated by the radiation reflected from the fiber Bragg grating during linear oscillations.

Since in this case, the change in ϵ during vibration occurred in the measurement range of ϵ at one wavelength, one dependence p(t) was sufficient to calculate the probability density of the distribution of ϵ(t) using Figure 11a. Therefore, an example of the histogram of the distribution of ϵ is not given in the work.

**Figure 13:**
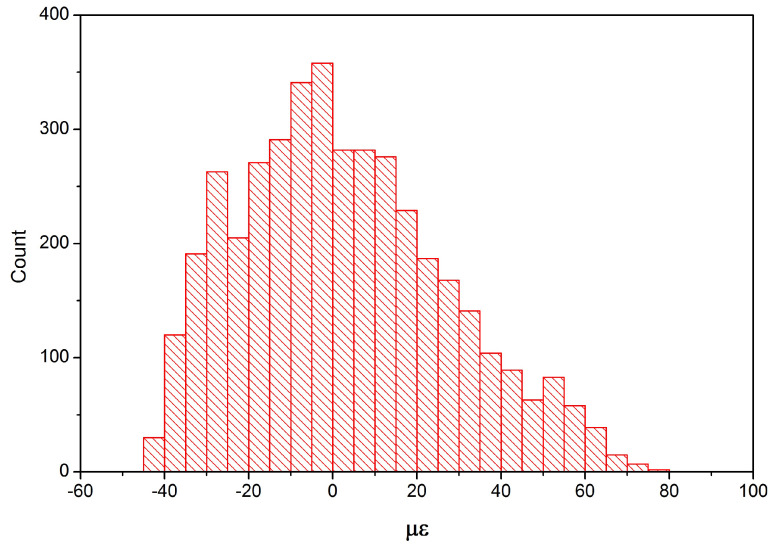
Distributions of the magnitude of the strain.

To increase the magnitude of the strain fluctuations, the flexible rods were attached to the rotating part of the device, in contact with the fixed elements of the experimental installation during rotation. This allowed us to increase the magnitude of ϵ(t) by more than two times, as well as to make the shape of the oscillations more complex. Similarly to the previous case, the dependencies p(t) of the power of the radiation reflected from the FBG at different temperatures of the semiconductor laser (respectively, at different wavelengths) were measured. The histogram of the distribution of ϵ was calculated using the method described above for the case of four wavelengths (Figure 13). The resulting distribution of ϵ was asymmetric, which was explained by the appearance of a bending of the fiber, which prevented the compression of the FBG when the zero value of ϵ was reached relative to the free (i.e., unloaded) state of the FBG during vibration.

## 7. Discussion

This paper considered a uni-axial deformation—a stretching or compression of the FBG. However, in most practical cases, in addition to stretching, there is a bending of the optical fiber with the FBG. A detailed consideration of the effect of deformation fields on the reflection spectra of Bragg gratings is given in [46,47], which can be taken into account in the calculations. However, the values of ϵ calculated according to the above method can be regarded as equivalent values of relative deformation.

Obviously, for strain measurements, it is necessary to ensure the stability of the wavelengths at which measurements are made. If a broadband radiation source, a spectral filter, and photodetector modules are used, which are constantly tuned to the same wavelength in the optical measurement scheme, then it is assumed that such a device ensures the stability of measurements. However, with a wide spectrum of the radiation source, the power coming from the filtered spectral ranges is significantly less than when using a narrow-band laser. Consequently, the measured dependencies p(t) have the worst signal-to-noise ratio, which has the strongest effect in the case of a wide frequency range of recorded signals. The problem of using standard components of WDM systems (semiconductor lasers, multiplexers, demultiplexers, and photodetectors) is the difficulty of matching the wavelengths of WDM elements with the reflection spectrum of the Bragg fiber lattice, since the latter depends on temperature. An even more difficult case for the use of WDM components are FBGs attached to or mounted in any structural elements, since the temperature coefficient of expansion in this case may be significantly higher than the FBG itself, which may require a significant change in the calculation algorithm.

When using a semiconductor laser, whose wavelength tuning is achieved by changing the temperature of the heterojunction, it is relevant to determine the requirement for the maximum allowable deviation $\Delta \lambda_{p}$ of the wavelength from the required one. This is most relevant for a method using a sequential change in the laser wavelength. If we assume that the condition $\Delta \lambda_{p}<<\sigma_{s}$ must be fulfilled, then in order to fulfill this condition, it is necessary to have:

(16) $$\Delta T_{p}\ll \sigma_{s}/\alpha_{T}$$

where $\Delta T_{p}$ is the maximum permissible temperature deviation from the required value during the measurement process, and $\alpha_{T}$ is the temperature coefficient of the laser wavelength change. As indicated above, for typical parameters of FBGs and DFB lasers, the estimated value is 0.1 °C, which is easily achievable.

However, if the temperature measurement uses a sensor built into the semiconductor laser housing (for example, a thermistor), and even more so if the sensor is mounted outside the laser housing, the temperature measured by the sensor may differ from the temperature of the laser heterojunction. This temperature difference can be caused both by the transient process of heating or cooling the laser structure when the temperature changes to adjust the wavelength and by the specifics of the thermal resistances of the structural elements of the Peltier element, the cooling radiator, and the laser, which retain some temperature difference in thermal equilibrium after the end of the transition process. If the calibration of the dependence of the laser wavelength on temperature was performed for a structure used for deformation measurements, then the presence of this temperature difference would not affect the methodology and accuracy of deformation measurement, since the calculated wavelengths according to the calibration dependence would correspond to the true value of the laser radiation wavelength. To eliminate the influence of the transient thermal process on the measurement accuracy, it is sufficient to start recording the signal from the photodetector after establishing thermal equilibrium, which is easy to determine by stabilizing both the measured temperature and the average value of p(t). The highest accuracy can be achieved by using a laser diode directly as a temperature sensor. However, the measuring device for this purpose is non-standard and requires calibration.

It is necessary to know the maximum value of $p_{max}$ at $\lambda_{LD}=\lambda_{FBG0}$ to determine the dependence of the strain value ϵ(t) on the dependencies p(t). In the above method, it was assumed that the spectral density of the radiation source was constant (i.e., either a broadband source or several lasers). In measurements with a sequential change in the wavelength of a semiconductor laser, it was also assumed that the laser radiation power was constant with a change in wavelength. However, when the temperature of a semiconductor laser changes, in addition to changing the wavelength, there may also be a change in the radiation power if the laser does not have thermal stabilization. If a semiconductor laser is equipped with a thermal stabilization system for the output radiation power, then power stabilization is performed by changing the laser current, which can also lead to a change in the laser radiation spectrum. Therefore, it is advisable to pre-measure the radiation spectrum of the selected semiconductor laser at different temperatures to obtain the calibration dependence $p_{max}\left(T\right)$ and the possibility of using it to determine the distribution of the strain ϵ(t) using a sequential change in the wavelength of the laser radiation. The semiconductor laser used in the experimental part of the work had a linear decrease in radiation power from temperature with the coefficient of 1.57% per degree Celsius. Therefore, when the histogram of the distribution of the magnitude of strain at the vibration (Figure 13) was determined, the described influence was neglected.

The possible measurement range of strain (Δϵ) can be calculated using the formula:

(17) $$\Delta \epsilon =m_{w}\sigma_{s}/k_{\epsilon }$$

where $m_{w}$ is the number of wavelengths used. The Δϵ range is estimated at 500μϵ for $m_{w}$ = 10 and $\sigma_{s}=0.05$ nm, which corresponds to the parameters of the FBG used in the experimental part of the work. An increase in the Δϵ range can be achieved either by increasing the number of wavelengths used simultaneously, or by using fiber Bragg gratings with a wider spectral reflection band. Presumably, achieving a Δϵ measurement range of 1000μϵ or more is not a technical problem when using standard fiber-optic components.

The paper considered two types of dependence of the reflection spectrum of fiber Bragg gratings: trapezoidal and Gaussian. Such dependencies can be assumed to be a fairly accurate approximation of typical reflection spectra of FBG, which allowed us to obtain analytical expressions for calculating the strain of FBG (i.e., ϵ(p)). However, the algorithm discussed above can also be applied to other types of reflection spectra of FBG, if there is an unambiguous correspondence between the wavelength and the magnitude of the spectral density in a certain wavelength range. In this case, the dependence of ϵ(p) can only be determined numerically.

## 8. Conclusions

The developed algorithm for determining changes in the strain ϵ(t) of objects during vibration based on simultaneously measured power dependencies p(t) of the radiation reflected from the FBG can be implemented for various forms of the spectrum of the radiation reflected from the FBG without the need to sort through all the elements of the accumulated data arrays during their processing. In particular, the numerical simulation method demonstrated the possibility of modeling p(t) signals detected by photodetectors at various wavelengths, and the subsequent restoration of the dependence ϵ(t) for the FBG and semiconductor lasers with typical parameters in the range up to ±1000 μϵ.

The numerical simulation method showed and experimentally confirmed the occurrence of the second harmonic of the signal recorded by the photodetector and the disappearance of the first harmonic when the resonant wavelength of the FBG in the undeformed state coincided with the wavelength of the laser radiation at the sinusoidal linear vibration of the FBG.

It was also shown that it was possible to determine the probability density of the distribution ϵ(t) for a stationary random process from the dependencies p(t) measured with successive changes in the wavelength of the radiation, which was confirmed by the results of the numerical simulation and experimental research.

The performed estimates showed that when using a semiconductor laser and a fiber Bragg grating with typical parameters, the accuracy of maintaining the laser temperature should be at least 0.1 °C for measurement at each wavelength.

## Data Availability

Data are contained within the article.
